# Childhood Environmental Instabilities and Their Behavioral Implications: A Machine Learning Approach to Studying Adverse Childhood Experiences

**DOI:** 10.3390/bs14060487

**Published:** 2024-06-08

**Authors:** Priscilla Mansah Codjoe, Nii Adjetey Tawiah, Daniel Alhassan

**Affiliations:** 1 College of Arts and Sciences, Southern Illinois University Edwardsville, Campus Box 1653, Edwardsville, IL 62026, USA; 2 College of Humanities, Education and Social Sciences, Delaware State University, 1200 N. DuPont Highway, Dover, DE 19901, USA; ntawiah@desu.edu; 3 Wells Fargo, 11625 N. Community Hse Rd, Charlotte, NC 28277, USA; danhmed@gmail.com

**Keywords:** adverse childhood experiences, residential mobility, family dynamics, childhood behavioral outcomes, machine learning

## Abstract

Adverse childhood experiences (ACEs) include a range of abusive, neglectful, and dysfunctional household behaviors that are strongly associated with long-term health problems, mental health conditions, and societal difficulties. The study aims to uncover significant factors influencing ACEs in children aged 0–17 years and to propose a predictive model that can be used to forecast the likelihood of ACEs in children. Machine learning models are applied to identify and analyze the relationships between several predictors and the occurrence of ACEs. Key performance metrics such as AUC, F1 score, recall, and precision are used to evaluate the predictive strength of different factors on ACEs. Family structures, especially non-traditional forms such as single parenting, and the frequency of relocating to a new address are determined as key predictors of ACEs. The final model, a neural network, achieved an AUC of 0.788, a precision score of 0.683, and a recall of 0.707, indicating its effectiveness in accurately identifying ACE cases. The model’s ROC and PR curves showed a high true positive rate for detecting children with two or more ACEs while also pointing to difficulties in classifying single ACE instances accurately. Furthermore, our analysis revealed the intricate relationship between the frequency of relocation and other predictive factors. The findings highlight the importance of familial and residential stability in children’s lives, with substantial implications for child welfare policies and interventions. The study emphasizes the need for targeted educational and healthcare support to promote the well-being and resilience of at-risk children.

## 1. Introduction

Adverse childhood experiences (ACEs) are distressing events that happen during a person’s early years and tend to have long-lasting effects on their lives [1]. ACEs have emerged as a critical focus in public health research due to their profound implications on long-term health and psychological outcomes. Since Felitti, Anda, and their team’s landmark study in 1998, the scope of ACEs research has expanded significantly, revealing deeper connections between early life stressors and a multitude of adverse adult outcomes [1,2].

ACEs encompass various forms of abuse, neglect, and household dysfunction, which have been consistently linked to chronic health issues, mental illnesses, and social challenges [3,4]. Research has shown that ACEs are more prevalent in certain demographics, notably among economically disadvantaged or minority communities [5]. While the cumulative effect of multiple ACEs and their exacerbation of health issues is well-documented [6,7], there is a growing recognition of the need for early identification and trauma-informed intervention [8].

Research on ACEs has identified a range of traumatic experiences, including physical, emotional, and sexual abuse, household dysfunction, and various forms of neglect. These experiences have been linked to a wide array of negative outcomes, such as increased risk of mental health disorders, substance abuse, chronic diseases, and socio-economic challenges [9,10]. Studies have also highlighted the cumulative nature of ACEs, where an increase in the number of adverse experiences correlates with a heightened risk of negative health outcomes [2,11]. The prevalence of ACEs is notably higher in populations facing socio-economic disadvantages, indicating a clear link between childhood adversity and later life inequalities [5,12].

While a considerable volume of research has examined the direct impacts of ACEs, less attention has been given to the predictive factors that might predispose individuals to these experiences. Predominantly, factors such as socioeconomic status, family history of mental health issues or substance abuse, and exposure to violence have been identified as primary predictors of ACEs [13,14]. However, these factors often provide a limited view, neglecting the potential influence of more subtle and interactive elements like family structure, parental education level, and frequency of relocations to a new address.

There exists a notable gap in the literature regarding the complex interplay of these less-explored predictors. While traditional studies have acknowledged the individual impact of factors such as family structure and economic conditions, the complex interactions between these factors and their collective influence on the incidence of ACEs remain underexplored [13,15,16]. To address this gap, our study employs advanced machine learning techniques to dissect the combined predictive power of family structure, parental education levels, and the frequency of relocations to a new address, as well as other predictors, in relation to ACEs. This novel approach allows for the exploration of intricate patterns and interactions that traditional statistical methods might overlook, offering a more refined understanding of these predictors and their synergistic effects on ACEs.

The purpose of this study is to employ a machine learning methodology in identifying key factors that impact ACEs in children aged 0–17 years and to develop a predictive model to estimate the likelihood of ACEs.

The article is structured as follows: Section 2 details the data sources and methodological approach, emphasizing the use of machine learning for comprehensive analysis. Section 3 presents the results, focusing on the nuanced effects of family structure, parental education, and relocation frequency on ACEs. Section 4 and Section 5 contain the discussion and limitation of study respectively. Finally, the conclusion in Section 6 discusses the implications of these findings for future research, policy development, and intervention strategies in the realm of childhood adversities.

## 2. Materials and Methods

### 2.1. Data and Source

This study utilizes a secondary dataset from the National Survey of Children’s Health (NSCH), specifically focusing on data from the years 2018–2019. This dataset provides extensive insights into various aspects of the health and well-being of children aged 0–17 years in the United States of America. It is publicly accessible (accessed on 29 September 2021) on the NSCH website: https://www.childhealthdata.org/learn-about-the-nsch/NSCH (accessed on 20 April 2024). Initially comprising 59,963 cases, the dataset was refined to 59,077 cases after excluding those with missing cases in the outcome. This sample forms the basis of our analysis. The NSCH is known for its robust methodologies, including random sampling, stratification, and weighting, to achieve a nationally representative sample. Detailed information on data collection methods can be found in the NSCH codebook, available here: https://www.childhealthdata.org/learn-about-the-nsch/nsch-codebooks (accessed on 20 April 2024). For this study, 70% of the sample, or 41,352 cases, was used as the training set (the subset of data used to train the machine learning model). The dataset was divided into training and test samples with stratification based on the outcome variable, ensuring balanced representation across both samples. The remaining 30%, or 17,725 cases, was used as the independent test sample (the subset of data used to evaluate the performance of the trained model).

### 2.2. Outcome: ACEs

The outcome variable in our study is the ACEs measure from the NSCH dataset. This variable categorizes ACEs into three levels based on the child’s exposure to one or more of the nine specified ACEs. These ACEs cover a range of experiences crucial for understanding their potential impact on a child’s well-being. They include family income challenges, parental separation or divorce, the death of a parent or guardian, the incarceration of a parent or guardian, exposure to domestic violence, victimization or witnessing of neighborhood violence, living with someone who has mental illness or substance abuse issues, and experiencing racial or ethnic discrimination. Each factor represents a significant aspect of a child’s environment that can influence their overall health and development.

### 2.3. Machine Learning Approach

In analyzing ACEs through machine learning techniques, we leveraged the capabilities of the tidymodels ecosystem in R [17]. This analysis was conducted using R version 4.3.2 [18]. The chosen machine learning algorithms were specifically selected for their proficiency in addressing the intricacies and unique characteristics of the ACEs dataset.

#### 2.3.1. Overview of Machine Learning Algorithms

In our investigation of ACEs, we employed a variety of machine learning algorithms, each selected for its unique strengths in addressing specific aspects of the dataset.

We began with Logistic Regression [19], a robust method for binary classification problems, which is crucial for estimating the likelihood of ACE occurrences based on various predictors. Additionally, the K-Nearest Neighbors (KNN) algorithm, applied through the kknn package [20], was utilized for its effectiveness in situations where feature similarity influences classification, which is particularly useful in cases with non-linear decision boundaries. In addition, the decision tree algorithm, from the rpart package [21], was incorporated for its clear interpretability and simplicity. This approach provides an accessible understanding of the decision-making processes within the data.

Furthermore, the random forest algorithm, using the randomForest package [22], was employed. This algorithm enhances prediction accuracy and stability by combining multiple decision trees and is particularly valuable in managing high-dimensional data. Also, AdaBoost, implemented via the C50 package [23], was included in our analysis. This technique effectively amalgamates weaker models to improve prediction precision by reducing bias and variance.

The study utilized extreme gradient boosting (XGBoost) [24], from the xgboost package [25], known for its exceptional performance in classification tasks and its capability to handle complex and unstructured datasets. Lastly, neural networks, facilitated by the nnet package [26], were chosen for their capacity to model complex non-linear relationships and discern intricate patterns within large datasets, which is particularly relevant in ACE research. Each algorithm was rigorously tuned for optimal performance, and a 10-fold repeated cross-validation scheme was employed to ensure the reliability and robustness of our models.

#### 2.3.2. Preprocessing

In the preprocessing stage of our machine learning approach, significant emphasis was placed on refining the quality of our training data to enhance the robustness of our analysis. A key procedure involved collapsing the rarest factor levels in the training samples [27]. This tactic aimed to minimize the noise associated with very rare factor levels while ensuring the preservation of at least two factor levels in our dataset. Implementing such a strategy is essential for maintaining the integrity and interpretability of our models, particularly given the diversity and potential sparsity of datasets in ACE studies.

Our initial assessment revealed approximately 0.05% missing data in ACE predictors and 0.01% missing data in the ACE outcome. All missing data in the outcome were removed. To address the missing data in the predictors, we implemented a strategic approach for imputation, which is vital for ensuring the dataset’s completeness and reliability. For numeric predictors, mean imputation was employed to preserve the central tendency of the data. In contrast, for categorical predictors, mode imputation was utilized, effectively maintaining the distributional characteristics of the variable by replacing missing values with the most frequent category.

#### 2.3.3. Hyperparameter Optimization

In our study, we conducted comprehensive hyperparameter tuning (the process of optimizing the settings that govern the training process of a machine learning model) for several predictive models, informed by contemporary machine learning methodologies [27]. For the decision tree model, the focus was on optimizing two key parameters: cost complexity (cost_complexity) and the minimum number of data points required at each node (min_n). In the random forest model, adjustments were made to the number of trees (trees) and the minimum size of nodes (min_n). Neural network models were fine-tuned by adjusting the regularization parameter (penalty) and the number of training epochs (epochs), ensuring a balance between model complexity and adequate training. The Gradient Boosting model’s tuning involved modifying the number of boosting rounds, tree depth (tree_depth), and learning rate (learn_rate), which are integral to guiding the model’s learning efficacy and capacity. For the K-Nearest Neighbors (KNN) model, the tuning concentrated on the number of neighbors (neighbors), and for the AdaBoost model, it involved the number of boosting rounds (trees). The hyperparameter optimization for each model was meticulously carried out with an emphasis on maximizing the AUC. A grid search strategy was employed to systematically navigate the defined parameter spaces. Details on the ranges of tuned hyperparameters can be found in the supplementary text.

#### 2.3.4. Application to Independent Test Sample

We refined all seven models by retraining them on the entirety of the training dataset. This retraining process was informed by the optimal hyperparameter settings, which were identified through a comprehensive 10-fold cross-validation approach. The objective was to accurately predict ACE levels in a completely independent test sample, thereby ensuring a distinct separation between the training and testing phases of the model.

### 2.4. Model Evaluation Metrics

In our comprehensive study, we employed a range of key performance metrics to evaluate the efficacy of machine learning models in categorizing ACEs. These metrics included the area under the receiver operating curve (AUC), F1 score, recall, precision, balanced accuracy, and the MCC. The AUC was pivotal in quantifying the model’s ability to differentiate between ACE categories, with higher values indicating superior discriminatory capabilities [28,29]. Precision was critical for accurately classifying each ACE category, particularly in identifying children with multiple ACEs [30]. Recall or sensitivity was equally important, measuring the model’s ability to correctly identify all instances of ACE exposure out of the actual cases of ACEs. This is crucial in not missing cases in children with multiple ACEs [31]. The F1 score, a harmonic mean of precision and recall, provided a balanced view of the model’s accuracy across ACE levels, ensuring unbiased performance toward any specific category. Balanced accuracy contributed valuable insights into the model’s performance across the ACE spectrum, especially in datasets with skewed ACE distributions [32]. The MCC gives a comprehensive evaluation of the model’s overall performance, accounting for all aspects of the confusion matrix. It is a robust measure of accuracy in all ACE categories [33]. These diverse metrics ensured a rigorous and multifaceted evaluation, recognizing the intricate nature of ACE categorization. For the “best” performing model, identified based on its superiority in most metrics, we further analyzed its receiver operating characteristic (ROC) curve and the AUC of the precision–recall curve. Employing the methodology suggested by [34] for multiclass problems, we used the One-versus-Rest (OvR) approach on both the ROC and the PR curves, providing a comprehensive view of the model’s performance across all ACE categories.

#### Feature Importance

After the best performing model (final model) is selected, we calculated the feature importance of the predictors using a permutation-based approach [35,36] based on a random sample of 1000 observations and 100 permutation rounds on each variable from the test data. This involves assessing the increase in prediction error after the values of a feature are randomly shuffled. This shuffling breaks the association between the feature and the outcome, thereby revealing its importance: the more the error increases, the more significant the feature is for the model’s predictions [35,37]. To ensure robustness, we calculated this importance using an independent test sample rather than the training sample, following best practices suggested in the literature [35]. To complement the understanding of feature influence, we utilized Accumulated Local Effect (ALE) plots and partial-dependence profile (PDP) plots [38] to describe ALE as a technique that visualizes the effects of features in black box models. This method is particularly valuable for handling correlated features, as it provides an unbiased view of their effects on the model’s output. Additionally, we incorporated PDP plots as outlined by [39]. The PDP plots, which are useful for visualizing the marginal effect of a feature on the predicted outcome, were averaged over the distribution of the other features. The feature importance, ALE, and PDP plots were obtained using the DALEX and DALEXtra R packages [40,41].

## 3. Results

### 3.1. Samples

Our study’s analysis of demographic and clinical characteristics in both the training and test samples provides a picture of the prevalence and diversity of ACEs and related factors.

In the training sample, a significant portion (61.49%) reported no exposure to ACEs, while 20.78% had experienced one ACE, and 17.73% encountered two or more ACEs. This distribution highlights the varied prevalence of ACEs, illustrating the different levels of exposure among participants. The test sample displayed a similar pattern, reflecting consistency in ACE prevalence across both groups. Notably, the mean number of relocations was slightly higher in the training sample (average 1.62, SD = 1.97) compared to the test sample, though this difference was not statistically significant. This pattern extended to the average number of children per household, with the training sample reporting slightly more children (average 1.86, SD = 0.87) than the test sample.

A significant difference emerged in household sizes, where the training sample averaged approximately 4.03 members (SD = 1.1), which was marginally larger than the test sample’s average of 4.01 members (SD = 1.1), with a *p*-value of 0.029. Variations in family composition ranged from single- or two-person households to those with six or more members in both samples, but a higher incidence of smaller households was noted in the test sample. Both samples showed a low prevalence of grade repetition among children (4.08% in the training sample), suggesting consistent academic progression. The age distribution was evenly spread across children aged 6–17 years in both groups. Parental health status was predominantly reported as excellent for both mothers (68.41% in training, 68.52% in test) and fathers (74.65% in training, 74.98% in test).

Household habits, such as smoking indoors, were uncommon (85.89% smoke-free in the training sample), and parental aggravation was rarely reported (95%, indicating infrequent occurrences in the training sample), indicative of low-stress family environments. The majority of children in both samples slept for the recommended duration appropriate for their age (69.17% in the training sample and 69.53% in the test sample), and a large proportion of families did not receive food or cash assistance (74.67% in training, 74.47% in test), pointing to economic stability. Neighborhood dynamics were similarly perceived in both samples, with 60.56% of the training sample and a comparable proportion in the test sample acknowledging supportive neighborhoods, and 69.38% in the training sample definitely agreeing on the safety of their neighborhoods.

These descriptive statistics offer a detailed comparison across various crucial predictors in both the training and test samples. The consistency observed in these characteristics underscores the robustness of our machine learning analysis, as illustrated in the figures in the supplementary text.

### 3.2. Selecting ML Model

Among the models analyzed, tree-based approaches such as random forest, adaptive boosting, and extreme gradient boosting demonstrated comparable performances across the six metrics. These models, known for their robustness and ability to handle complex datasets, showed a consistent level of accuracy and predictive power in identifying ACEs (Table 1). However, the decision tree model stood out for its notably lower performance, particularly in terms of the AUC and accuracy. The standout model in our analysis was the neural network, which achieved the highest scores across several key metrics. It recorded the best AUC at 0.788, demonstrating its superior ability to distinguish between different ACE categories. Additionally, it achieved the highest recall (0.707) and precision (0.683) scores, indicating its effectiveness in correctly identifying ACE cases and its reliability in positive predictions, respectively. The model also excelled in accuracy (0.708) and the Matthews Correlation Coefficient (MCC) (0.451), further underscoring its overall predictive strength. Interestingly, the neural network and the extreme gradient boosting model shared the same performance level in terms of the F1 score (0.68). These findings underscore the neural network model’s suitability as the “best” performing model for this dataset, given its superior performance across most metrics.

### 3.3. Prediction of ACEs

The ROC curve for children who had experienced two or more ACEs indicates a high true positive rate and a low false positive rate, underscoring the model’s robust performance in accurately distinguishing these more complex cases from other categories. This effectiveness, however, slightly diminishes when predicting the “No ACE” class, where the model still demonstrates solid effectiveness but with a marginally reduced discriminative ability compared to the “Experienced 2 or more ACEs” category.

**Figure 1:**
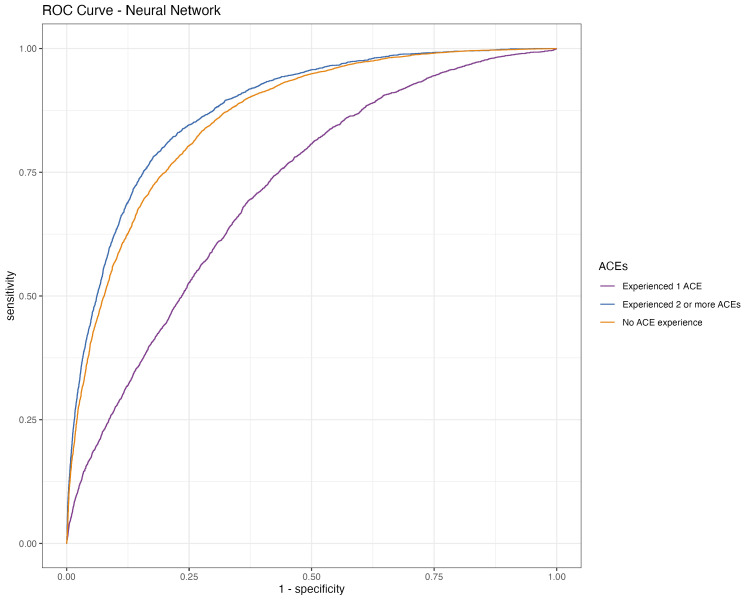
ROC curve of the best performing model, the neural network.

**Figure 2:**
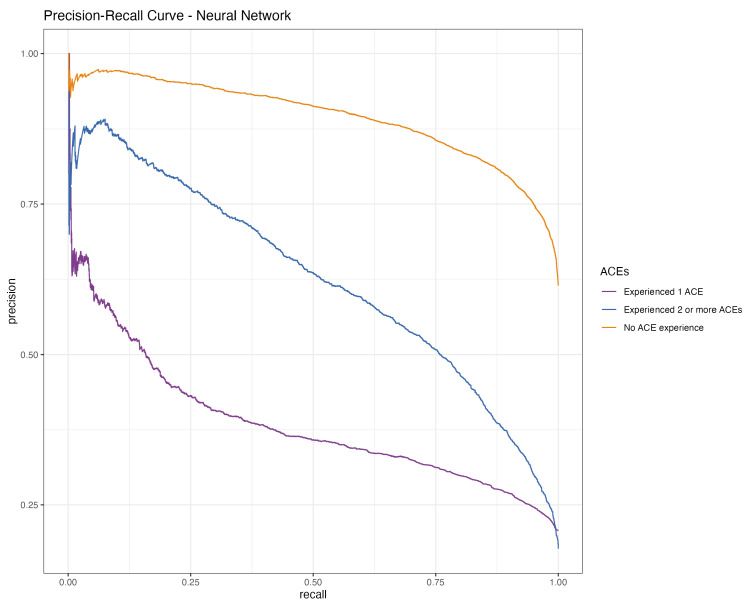
PR curve of the neural network.

The model encounters notable challenges in the “Experienced 1 ACE” category, as evidenced by a lower true positive rate and a higher false positive rate relative to the other classes. This finding suggests a potential area for improvement in the model’s ability to accurately classify instances of a single ACE (see Figure 1). The PR curve further shows high precision across all recall levels for the “No ACE” class, indicating a confident classification of cases with no ACE exposure against those with one or more ACEs. However, for the “Experienced 1 ACE” class, the model begins with high precision, which decreases as recall increases, implying that the model, in its efforts to include more true positives, starts to inaccurately classify some instances from other categories as “Experienced 1 ACE”. A similar trend is observed in the “Experienced 2 or more ACEs” class, where the precision notably decreases with rising recall, reflecting the model’s challenge in consistently and accurately identifying multiple ACE exposures (see Figure 2).

**Figure 3:**
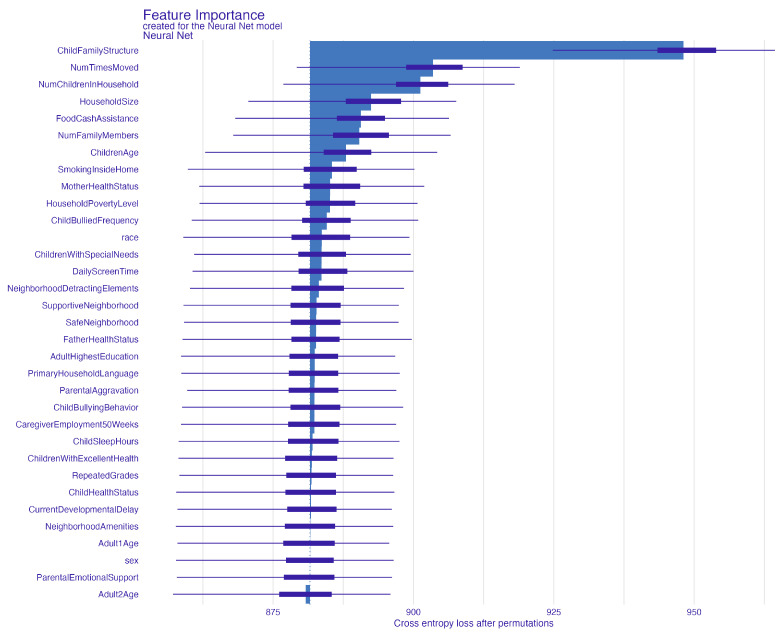
Permutation-based feature importance for the neural network model.

**Figure 4:**
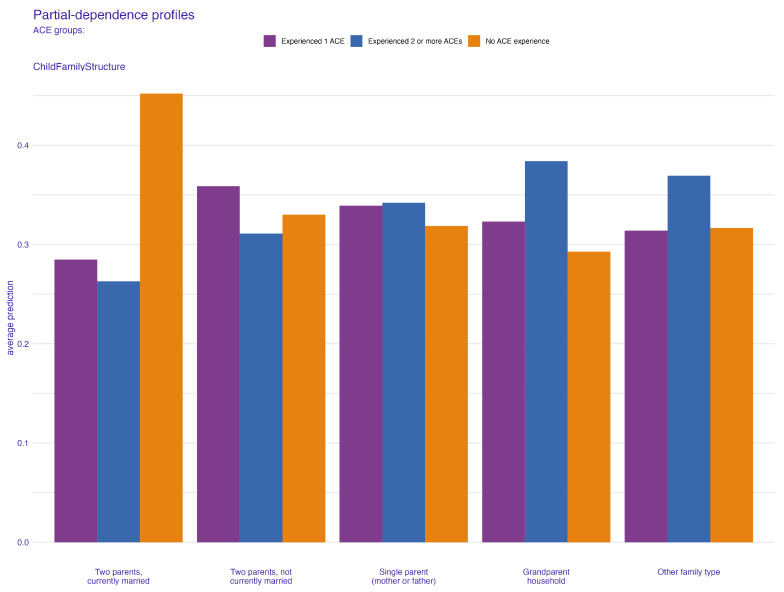
Partial-dependence profile plot for child family structure.

**Figure 5:**
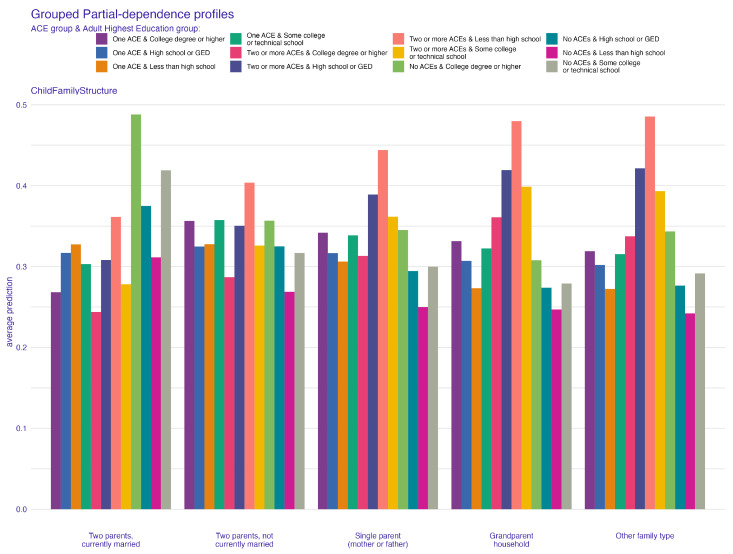
Partial-dependence profile plot for child family structure grouped by highest adult education.

In Figure 3, the permutation feature importance indicates the paramount role of the child’s family structure in ensuring robust predictions in the independent test sample, as denoted by a mean cross entropy value of 948. Uncertainty related to the application of 100 permutations is represented in the boxplot. Subsequent to family structure, the factor most affecting average prediction accuracy is how often the child has changed addresses. The count of children in the household, household size, and dependence on food and cash assistance are also key predictors, listed in order of their importance. To further elucidate how these important predictors specifically influence ACE prediction, we turn to PDP and ALE plots, focusing on the two most influential factors. These factors also feature prominently in the top five of our next best model, the extreme gradient boosting, reaffirming their predictive power. Figure 4 and Figure 5 present the PDP plots for the child’s family structure. Our analysis reveals varied contributions of different family types to the prediction of ACEs. A two-parent married household significantly predicts a lower likelihood of ACEs, as depicted in Figure 4. Conversely, families with two parents who are not married, single parents, or other family structures tend to predict higher ACEs. Further stratification by the parents’ highest education level in Figure 5 highlights intricate variations in predictions. Notably, in households where both parents are married, higher educational attainment correlates with a lower prediction of ACEs. However, for families with less educated single parents or other family structures, the likelihood of predicting one or more ACEs increases.

**Figure 6:**
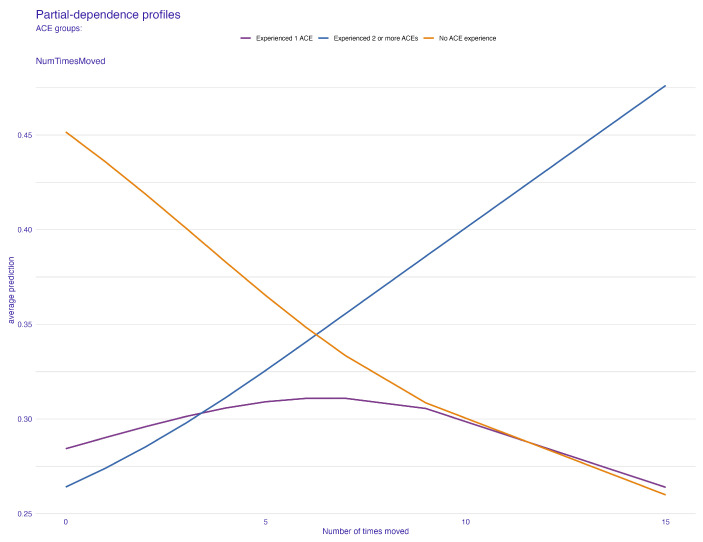
Partial-dependence profile plot for the number of times a child changed address.

**Figure 7:**
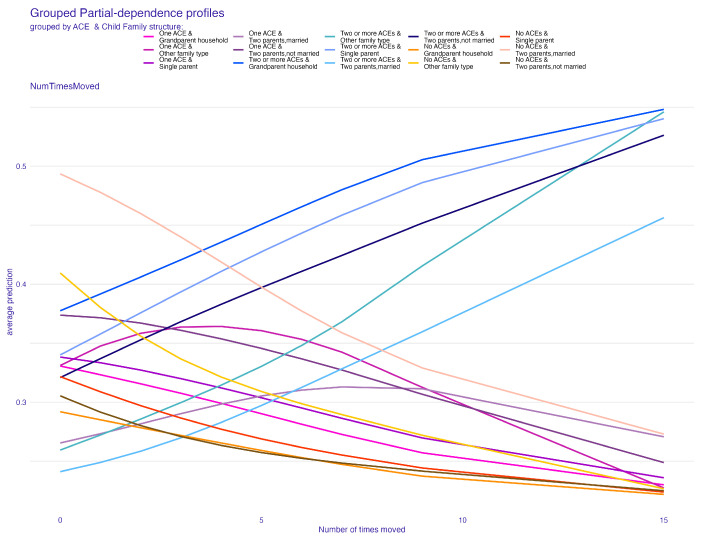
Partial-dependence profile plot for the number of times a child changed address grouped by the child’s family structure.

Figure 6 and Figure 7 present the PDP plots for the number of times a child changed address. Figure 6 reveals that a higher number of times a child changed address is a greater predictor of children who experienced two or more ACEs. As one will suggest, there is a negative relationship between the number of times a child changed address and the average prediction of the no-ACE effect. In other words, if a child has changed homes more times, it becomes less and less likely for that predictor to contribute importantly to predicting the group with no ACEs. The number of times a child changed address did not seem to affect the average prediction for predicting the likelihood of having one ACE.

**Figure 8:**
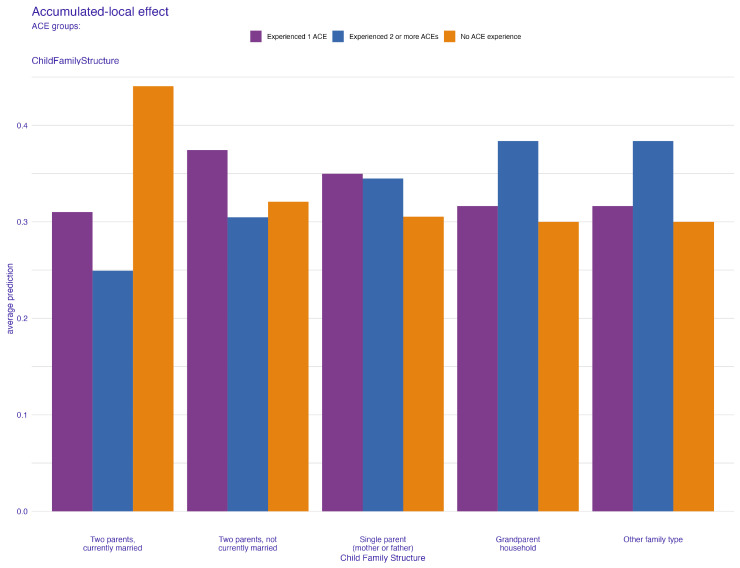
ALE plot for child family structure.

**Figure 9:**
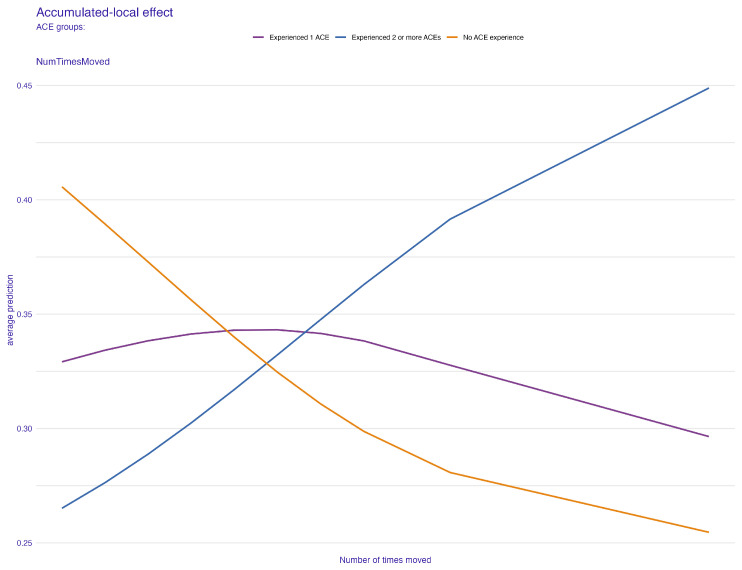
ALE plot for the number of times a child changed address.

To comprehensively understand the effects of our predictive factors, we further examined the ALE plots. In the context of the child’s family structure, Figure 8 reveals a pattern that is broadly consistent with the one observed in Figure 4 from the PDP plot. However, a notable difference emerges within the “other family type” group. This discrepancy suggests a relatively low correlation between the child’s family structure and another predictor in the model. This observation is pivotal as it indicates the uniqueness of the “other family type” group’s influence on ACE predictions, potentially hinting at complex dynamics within this category that are not as pronounced in more traditional family structures. Similarly, for the number of times a child changed address, or the frequency of relocations, the ALE plot (Figure 9) provides additional insights. It indicates a moderate correlation between the frequency of relocations and another predictor, compared to the pattern observed in Figure 6. This correlation is significant as it highlights the complex relationship between the instability of a child’s living situation and other factors that might contribute to ACEs.

## 4. Discussion

The present study’s exploration of the predictive power of ACEs, particularly emphasizing the roles of family structure and frequency of relocations, reveals profound insights into the dynamics that shape a child’s developmental environment. Our machine-learning-based approach not only underscores the predictive significance of these factors but also aligns with a body of existing empirical research that draws similar conclusions about their impact.

### 4.1. Family Structure and Its Impact on ACEs

The finding that family structure emerges as a robust predictor of ACEs resonates with the work of [42], who emphasize the vulnerability of children in non-traditional family settings to adverse experiences. Specifically, our results regarding children living in single-parent households align with the findings of [43], who reported an increased likelihood of experiencing environmental stressors and emotional neglect in such settings. Furthermore, the role of economic hardship, as discussed by [44], in exacerbating ACEs in single-parent and non-married parent families cannot be overstated. These economic challenges often translate into reduced emotional and developmental support for children, thereby increasing their ACE scores. Conversely, the lower likelihood of ACEs in two-parent married households, as shown in our findings, is consistent with studies emphasizing the benefits of a stable, two-parent environment in terms of emotional and economic support [43,45].

### 4.2. The Role of Frequent Relocations

The significant predictive value of the frequency of relocations highlights the profound impact of environmental instability on children. Frequent moves can disrupt children’s sense of security and attachment, contributing to emotional and behavioral issues. This aligns with the research by [46], who found that residential moves, particularly during critical developmental periods, can have lasting adverse effects on children’s emotional well-being. In fact, [47] further supports this by identifying a direct correlation between geographic mobility and emotional and behavioral adjustment difficulties in children. Additionally, [48] found that frequent relocation was linked to increased rates across all measures of child dysfunction. With frequent relocations come several school changes, which cause social disruption in the lives of the children. This can be particularly detrimental, impairing children’s ability to form lasting, supportive relationships [47].

These insights into the predictive power of family structure and relocation frequency can guide clinicians and social scientists in developing more targeted interventions and support systems. Understanding these key predictors allows for the stratification of risk and tailoring of preventive measures, an approach supported by the longitudinal research of [49,50]. For instance, enhanced support for children in high-risk family situations or those experiencing frequent relocations could mitigate the long-term impacts of ACEs.

## 5. Limitations of the Study

A key limitation of this study stems from its reliance on data from the NSCH. The use of self-reported information can introduce biases, notably recall and social desirability biases, which may affect the accuracy of reported ACEs [51]. Additionally, the NSCH’s variable scope, while extensive, might not capture all nuanced factors influencing ACEs, such as detailed family dynamics or specific cultural influences. This limitation is echoed in research emphasizing the need for diverse and longitudinal data for comprehensive child health analysis [52]. Moreover, the survey’s generalizability to specific sub-populations or different socio-cultural contexts could be limited, suggesting the need for caution in the broader application of the findings.

## 6. Conclusions

This study’s exploration of the predictive factors of ACEs using advanced machine learning techniques has uncovered significant insights into the roles of family structure, parental education levels, and the frequency of relocations. Our findings not only reinforce the complexity of the factors contributing to ACEs but also highlight the complex interactions between these elements. The use of machine learning has enabled a more in-depth analysis of these interactions, revealing patterns that traditional statistical methods may overlook.

Future research should continue the trajectory set by this study, aiming to refine predictive models of ACEs further. Incorporating a broader range of variables, cultural background, and access to community resources is imperative for a more comprehensive understanding of ACEs [53]. Additionally, exploring the impact of emerging factors, such as digital media exposure and changing social norms, could provide valuable insights into the evolving landscape of childhood experiences. Longitudinal studies would be particularly beneficial in understanding the long-term effects of these predictors on individuals’ life trajectories.

From a policy perspective, the findings of this study underscore the need for developing targeted support programs in educational and healthcare settings. Such programs should be designed to mitigate the impact of identified risk factors on children’s well-being. This could include enhanced training for educators and healthcare providers in recognizing and addressing the signs of ACEs, as well as the implementation of comprehensive support systems for at-risk families.

Moreover, our study advocates for greater community engagement in preventing and addressing ACEs. Community-based initiatives, such as parenting support programs and awareness campaigns, can play a crucial role in creating environments that nurture and protect children. Engaging community leaders and stakeholders in these efforts can help ensure that interventions are culturally sensitive and effectively address the unique needs of different communities.

In conclusion, the application of machine learning in ACE research represents a significant advancement in our understanding of the multifaceted nature of these experiences. By continuing to explore and refine these predictive models, we can contribute to the development of more effective strategies for preventing and mitigating the impact of ACEs. Ultimately, the goal is to foster a society where all children have the opportunity to grow and develop in a healthy and supportive environment.

## Figures and Tables

**Table 1:** Comparison of performance metrics across the seven models.

Model	AUC	F1 Score	Recall	Precision	Accuracy	MCC
Logistic Regression	0.783	0.680	0.704	0.674	0.701	0.440
KNN	0.710	0.632	0.652	0.621	0.641	0.323
Decision tree	0.500	0.762	0.615	0.615	0.500	-
Random forest	0.768	0.676	0.700	0.668	0.689	0.425
Adaptive Boosting	0.757	0.669	0.686	0.661	0.685	0.405
XGBoost	0.785	0.688	0.704	0.681	0.706	0.444
Neural Network	0.788	0.687	0.707	0.683	0.708	0.451

## Data Availability

Data are contained within the article and Supplementary Materials.

## Supplementary Materials

The following supporting information can be downloaded at: https://www.mdpi.com/article/10.3390/bs14060487/s1.
