# Supplementary material for: Childhood Environmental Instabilities and Their Behavioral Implications: A Machine Learning Approach to Studying Adverse Childhood Experiences
Source: Behav Sci (Basel). 2024 Jun 8;14(6):487. doi: 10.3390/bs14060487 (PMC11200692; doi:10.3390/bs14060487)
Supplement: Supplementary file 1 [file behavsci-14-00487-s001.zip › behavsci-2998940-supplementary.pdf]

Childhood Environmental Instabilities and Their Behavioral Implications: A Machine Learning Approach to Studying Adverse Childhood Experiences
Supplementary Text

Priscilla Mansah Codjoe   Nii Adjetey Tawiah   Daniel Alhassan

1 Results from Statistical Analysis of Train and Test sample data

The following output represents the results from the statistical analysis of train and test sample data.

Figure S1: These plots show the distributions of the features in the training and test sample with chi-square statistics for categorical features and t statistics for numeric features and the respective p-values.

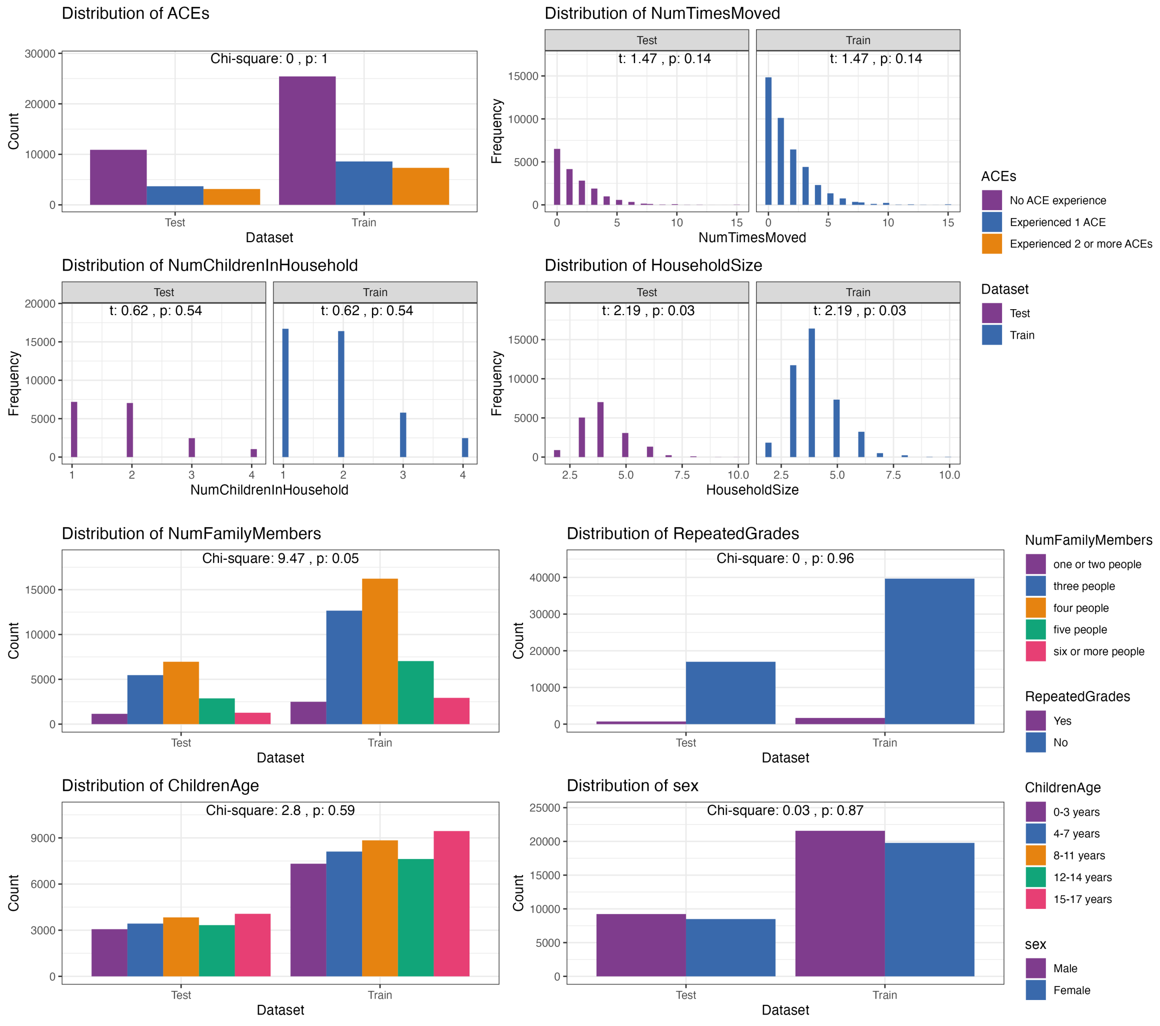

Figure S2: These plots show the distributions of the features in the training and test sample with chi-square statistics for categorical features and t statistics for numeric features and the respective p-values.

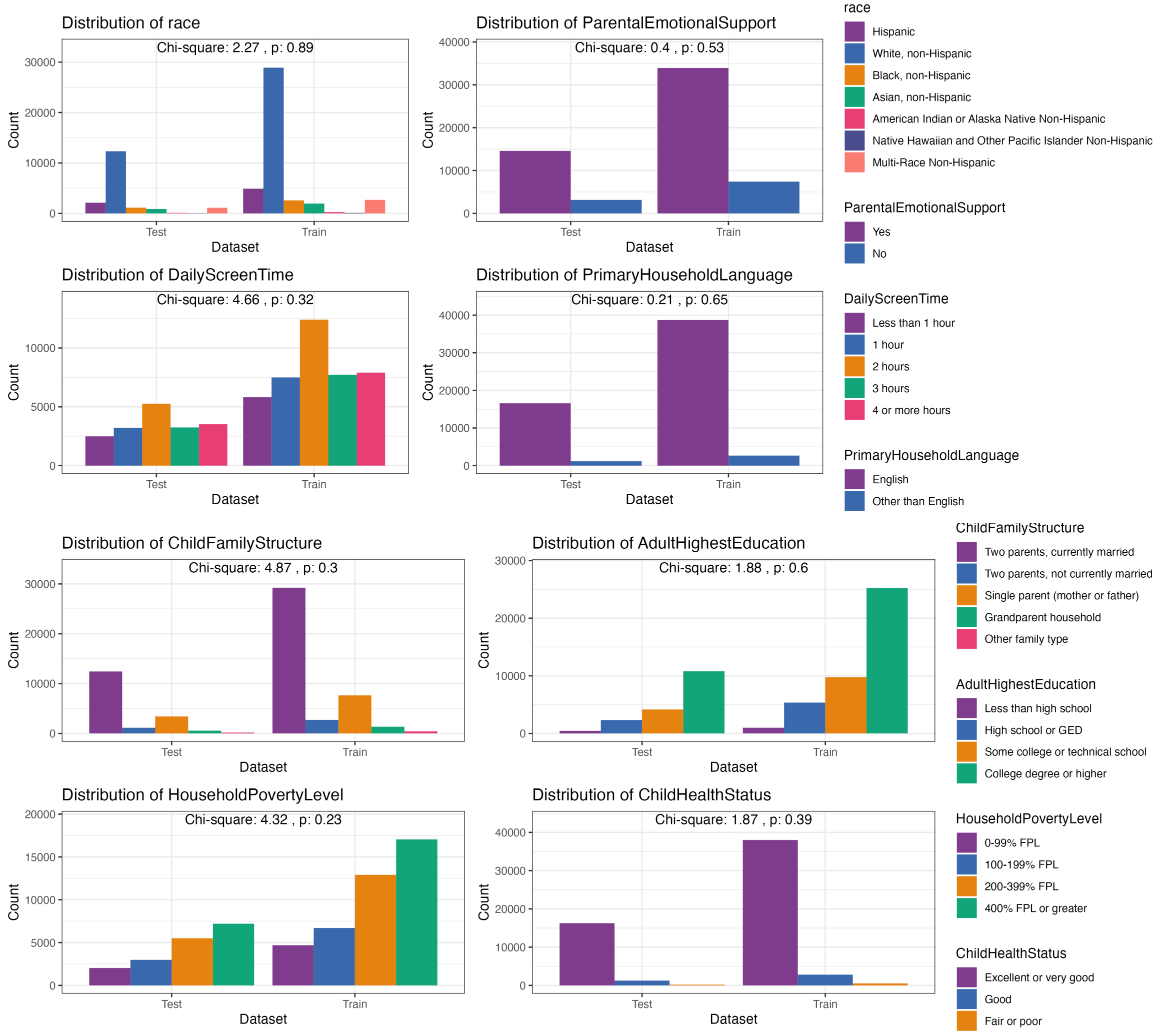

Figure S3: These plots show the distributions of features in the training and test sample with chi-square statistics for categorical features and t statistics for numeric features and the respective p-values.

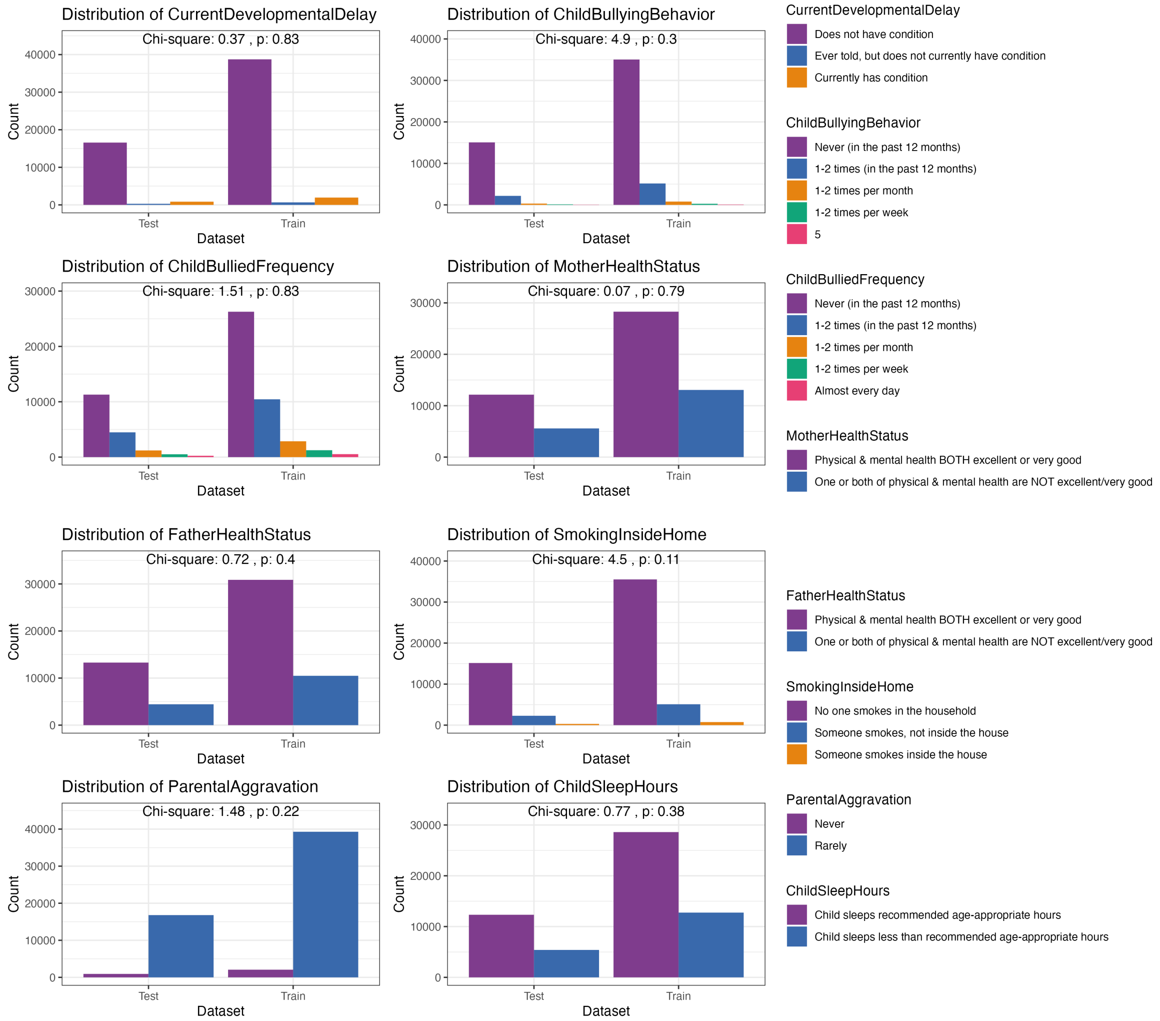

Figure S4: These plots show the distributions of the last 12 variables in the training and test sample with chi-square statistics for categorical features and t statistics for numeric features and the respective p-values.

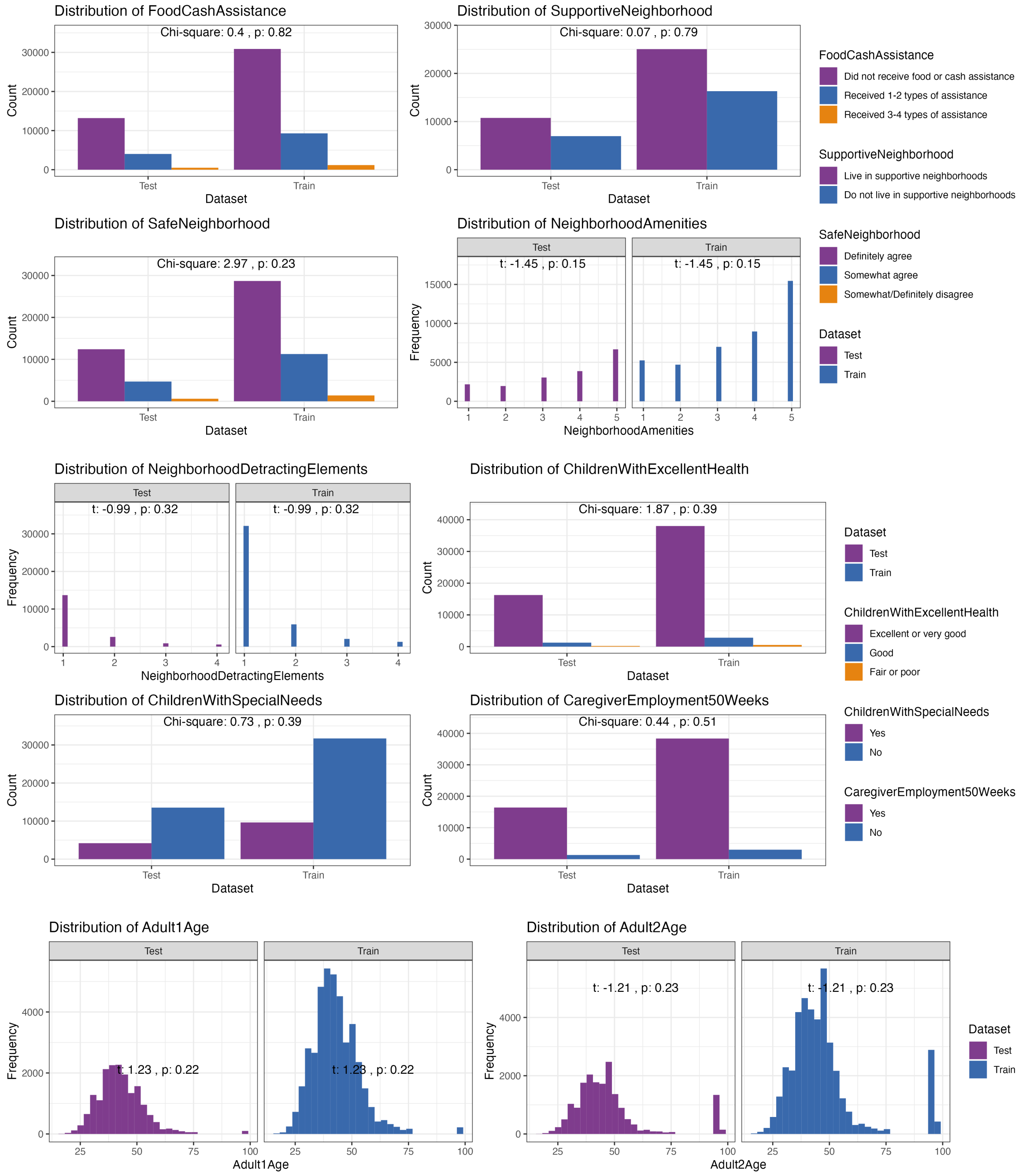

Table S1: Hyperparameters and ranges explored for each model

**Model**	**Range of hyperparameters explored**
Logistic Regression	Not applicable
Decision Trees	cost_complexity: [0.001, 0.01], min_n: [1, 10]
Random Forest	trees: [50, 100], min_n: [1, 10]
Neural Networks	penalty: [0.001, 0.1], epochs: [50, 100]
Gradient Boosting	trees: [50, 100], tree_depth: [1, 7], learn_rate: [0.01, 0.1]
KNN	neighbors: [1, 20]
AdaBoost	trees: [50, 100]
